# Development of a Portable Near-Infrared Spectroscopy Tool for Detecting Freshness of Commercial Packaged Pork

**DOI:** 10.3390/foods11233808

**Published:** 2022-11-25

**Authors:** Eduardo Arias, Verónica Sierra, Natalia Prado, Pelayo González, Giovani Fiorentini, Juan Díaz, Mamen Oliván

**Affiliations:** 1 Área de Sistemas de Producción Animal, Servicio Regional de Investigación y Desarrollo Agroalimentario (SERIDA), Ctra. AS-267, PK 19, 33300 Villaviciosa, Spain; 2 Asociación de Investigación de Industrias Cárnicas del Principado de Asturias (ASINCAR), Polígono de la Barreda, Parcela 1, 33180 Noreña, Spain; 3 Department of Animal Science, Campus Capao do Leao, Federal University of Pelotas, Pelotas 96010-970, Brazil

**Keywords:** near-infrared spectroscopy (NIR), modified atmospheres (MAP), microbiology, quality control, total viable counts (TVC), *Enterobacteriaceae*, lactic acid bacteria (LAB)

## Abstract

Real-time monitoring of meat quality requires fast, accurate, low-cost, and non-destructive analytical methods that can be used throughout the entire production chain, including the packaged product. The aim of this work was to evaluate the potential of a portable near-infrared (NIR) spectroscopy tool for the on-site detection of freshness of pork loin fillets in modified atmosphere packaging (MAP) stored on display counters. Pork loin slices were sealed in MAP trays under two proportions of O_2_/CO_2_/N_2_: High-Ox-MAP (30/40/30) and Low-Ox-MAP (5/20/75). Changes in pH, color, thiobarbituric acid reactive substances (TBARS), Warner–Bratzler shear force (WBSF), and microbiology (total viable counts, *Enteriobacteriaceae*, and lactic acid bacteria) were monitored over 15 days post-mortem at 4 °C. VIS-NIR spectra were collected from pork fillets before (through the film cover) and after opening the trays (directly on the meat surface) with a portable LABSPEC 5000 NIR system in diffuse reflectance mode (350–2500 nm). Quantitative NIR models by partial least squares regression (PLSR) showed a promising prediction ability for meat color (L*, a*, C*, and h*) and microbiological variables (R^2^_VAL_ > 0.72 and RPD_VAL_ > 2). In addition, qualitative models using PLS discriminant analysis obtained good accuracy (over 90%) for classifying pork samples as fresh (acceptable for consumption) or spoiled (not acceptable) based on their microbiological counts. VIS-NIR spectroscopy allows rapid evaluation of product quality and shelf life and could be used for on-site control of pork quality.

## 1. Introduction

Meat quality is a complex concept usually defined in terms of a set of attributes (appearance, color, water-holding capacity, lipid stability, microbial quality, texture, tenderness, juiciness, flavor, etc.) that determine whether meat can be consumed fresh or stored without deterioration for a reasonable period of time [1].

Preserving meat throughout maturation is essential to ensure safety, prolong shelf life, and maintain quality attributes [2]. A properly designed packaging method is required to maintain meat quality and provide convenience and ease of use for consumers [3]. Recently, the practice of butchers cutting meat on demand has been reduced and replaced by meat products being packaged and displayed in case-ready forms [4].

One of the most popular packaging methods is modified atmosphere packaging (MAP), in which the gaseous environment is removed and replaced by a desired gaseous atmosphere used to preserve fresh meat and maintain an attractive color. This type of packaging allows for an attractive presentation of meat products (sliced and packaged), resulting in convenience for consumers based on current purchasing habits [5,6]. However, maintaining the shelf life of meat, even with MAP, is still limited, as it is conditioned by microbiological growth, dehydration, discoloration, nutrient loss, texture changes, and lipid/protein oxidation [4,7]. Official methods for controlling bacterial spoilage during meat processing are based on culturing techniques and rely on regulatory inspections and intensive sampling regimes, which, while accurate, are not suitable for real-time monitoring of microbial contamination. For these reasons, there is a need to develop rapid, accurate, low-cost, and non-destructive mechanisms for monitoring the shelf-life of packaged meat that will allow efficient management of meat trays during marketing to avoid food waste and to ensure safety compliance.

Visible and near-infrared reflectance (VIS-NIR) spectroscopy provides informative spectral fingerprints of food samples and can capture changes that occur during storage. Therefore, it has been widely applied in food analysis as an alternative to traditional methods, as it is rapid, easy to perform, and does not require sample preparation or the use of chemical reagents [8,9].

The ability of NIR spectroscopy to predict the quality attributes of complex muscle foods, such as chemical composition, fatty acid profile, color and pigment content, water-holding capacity, tenderness, sensory acceptability, and other properties, has been widely demonstrated [10,11,12]. With respect to microbiological control, several authors found that NIR spectroscopy can be used to study changes in microbial loads (quantitatively or qualitatively) over a storage period [2,13,14,15,16,17]. In addition, one of the most interesting advantages of NIR spectroscopy for monitoring of meat shelf life is the fact that this technology allows the measurement of intact meat through plastic food packaging, as has been demonstrated with dry cured pork loin and chicken breast [6,14]. Although this property of NIR spectroscopy is very interesting, to our knowledge there has been no scientific study addressing the use of NIR spectroscopy for non-destructive determination of the freshness and quality of MAP pork fillets without having to open the trays at commercial counters. Moreover, the availability of portable NIRS instruments facilitates the use of a powerful tool to implement accurate and convenient control throughout the meat supply chain, enabling the analysis of large number of samples and real-time decision-making. Thus, this technology is extremely useful for controlling meat deterioration during production.

Having all of this in mind, the objective of this work was to evaluate the potential of VIS-NIR spectroscopy (350–2500 nm) for on-site prediction of meat quality (pH, color, texture, oxidation) and safety (microbial spoilage) by direct collection of spectral data on pork loin slices in MAP.

## 2. Materials and Methods

### 2.1. Meat Samples and Treatments

A total of 756 pork loin slices (0.5 cm thick) were collected 24 h post-slaughter from the *Longissimus thoracis et lumborum* (LTL) muscle of the right and left sides of 9 commercial male pig carcasses (crosses of (Large white × Landrace) × Duroc), that were slaughtered at 100–120 kg in 3 batches (3 animals each). Slaughtering was performed at a commercial abattoir following approved EU procedures.

After slicing the muscle, fillets (3 slices per tray, simulating commercial conditions) were placed in polystyrene INfresh MAP trays (Linpac, Pravia, Spain) covered with LINtop Star (Linpac, Pravia, Spain) high-barrier lidding film with O_2_ permeability lower than 5 cm^3^/m^2^/24 h/bar at 23 °C and 0% relative humidity, and packed under two MAP conditions with different proportions of O_2_/CO_2_/N_2_: High-Ox-MAP (30/40/30) and Low-Ox-MAP (5/20/75). These gas mixtures were from Freshline™ 3MIX (Air Liquid, Spain). The packing machine used was a MULTIVAC T-200 semi-automatic tray sealer (Multivac Inc., Kansas City, MO, USA) connected to a gas flushing system.

Meat trays were kept at 4 °C and analyzed at 7 storage times (1, 3, 5, 7, 9, 12, and 15 days), so the experimental design included a total of 252 samples (9 animals × 7 times × 2 MAP × 2 tray replicates). After the corresponding aging period, near-infrared (NIR) spectra were collected on intact trays through the barrier film. The atmosphere of the trays was checked for gas leakage with the OXYBABY^®^ M+ O_2_/CO_2_ portable digital headspace gas analyzer (WITT-Gasetechnik GmbH & Co. KG, Witten, Germany). This analyzer measures oxygen and carbon dioxide concentrations in the headspace of the package prior to opening the tray through a septum glued onto the packaged surface using the analyzer needle. Afterwards, trays were opened and meat samples were collected under aseptic conditions for microbiological analysis. NIR spectral collection and color measurements were performed directly on the meat surface after 15 min of blooming. Then, pH was measured, and a 20 g portion of meat was taken for TBARS determination. Finally, fillets were vacuum-packed in 20 μm polyamide/70 μm polyethylene bags, frozen, and stored at −20 °C until Warner–Bratzler shear force (WBSF) analysis.

### 2.2. Microbiological Analysis

For microbiological analysis, meat samples were processed according to ISO 7218 (International Organization for Standardization, 2007). Once the MAP trays were opened, meat samples of approximately 10 g were excised and transferred aseptically with sterile tweezers into a masticator bag, weighed, and homogenized with 90 mL of sterile (0.1%) buffered peptone water solution (Oxoid, Unipath Ltd., Basingstoke, UK) in a gravimetric diluter (Dilumat 3, AES, Chemunex, Bruz, France). The mixture was homogenized in a stomacher (IUL Instruments, Barcelona, Spain) for 2 min. For microbial counts, appropriate decimal dilutions of each sample were prepared and placed on the corresponding medium in Petri dishes. Total viable counts (TVCs) were determined on Plate Count Agar (PCA; Oxoid, Unipath Ltd., Basingstoke, UK) incubated at 30 °C for 72 h (ISO 4833-2:2013). *Enterobacteriaceae* were determined on Violet Red Bile Glucose Agar (VRBG, Merck, Darmstadt, Germany) after incubation at 37 °C for 24 h (ISO 21528-2:2017). Lactic acid bacteria (LAB) were determined on de Man, Rogosa, Sharpe medium Agar (MRS; Oxoid, Unipath Ltd., Basingstoke, UK) after incubation at 30 °C for 72 h (ISO 15214-1998). All plates were counted, and data were transformed into logarithms of the number of colony-forming units per gram of sample (log cfu/g).

### 2.3. Meat Quality Traits

Meat color on three 8 mm diameter spots of the exposed surface of pork fillets in each tray was measured with a Minolta CM-2300d spectrophotometer (Konica Minolta Holdings Inc., Osaka, Japan) with D65 illuminant and 10° standard observer angle, and zero and white calibration in the CIELAB space. Indicators of lightness (L*), redness (a*), and yellowness (b*) were measured after 60 min of blooming. Chroma (C*) and hue angle (h*) were calculated according to the following equations: C* = √ (a*^2^ + b*^2^) and h* = tan^−1^b*/a*, respectively [18].

Measurements of pH were performed using a penetration electrode and a portable pH meter (Mettler-Toledo Int. Inc., Reppenstedt, Germany).

Meat toughness was measured by the Warner–Bratzler shear force (WBSF) test. Fillets were thawed overnight and cooked at 75 °C for 30 min by immersion in a water bath. Eight cores (0.5 cm^2^ in cross-section) were extracted and cut perpendicularly by a WB shear blade set with a “V” slot blade (HDP/WBV) using the TA.XT Plus instrument (Stable Micro Systems, London, UK). The maximum load (kg) required for total split was recorded. Outliers in the results were detected by box plot and extreme values were deleted. Results are expressed as the mean WBSF maximum load (kg) for each sample.

Thiobarbituric acid reactive substances (TBARS; mg malonaldehyde/kg muscle) were measured according to the method of Botsoglou et al. [19] using derivative spectrophotometry.

### 2.4. NIR Spectroscopy

#### 2.4.1. Spectral Collection and Pre-Treatment

Diffuse reflectance spectra (350–2500 nm) were collected on the pork slices through the plastic film of the intact trays and also directly on the meat surface, after opening the trays, using a portable VIS/NIR LabSpec^®^ 5000 NIR spectrometer (ASD Inc., Boulder, CO, USA) equipped with an ASD fiber optic contact probe (21 mm window diameter). Instrument control and initial spectral manipulation were performed using the Indico Pro software package (ASD Inc., Boulder, CO, USA). Prior to spectral acquisition, the instrument was calibrated using a Spectralon tile as the white reference. In each meat tray, 5 spectra (40 scans per spectrum collected within the scanning area of the probe head) were taken at different locations on the pork surface, to increase the area of meat surface scanned and reduce the sampling error [20]. These spectra were visually examined for consistency (removing spectral outliers) and then averaged. Afterwards, the average of 2 meat trays (replicates) for each studied aging point was calculated, resulting in a final population of 126 spectral data points.

The data were subsequently imported into Unscrambler X v.10.5 (CAMO^®^, Trondheim, Norway) for chemometric analysis. To develop NIR models, the data were divided into a calibration dataset (*n* = 106) and a validation dataset (*n* = 20). The data split between calibration and validation subsets (approximately 85 and 15% of total samples) was carried out by manual and random selection to ensure representation in each subset of samples from all factors (MAP type and post-mortem time) in order to maximize variability in both calibration and validation sample tests.

Outlier spectra were identified during model development and removed based on the following criteria: studentized residuals higher than 2 and leverage higher than 3 times the average leverage, calculated according to Faber [21].

For chemometric analysis, spectra were transformed from reflectance to absorbance (log 1/R) and pre-processed in order to reduce scattering effects and heighten the signals related to the organic compounds using various methods, including standard normal variate (SNV), detrend correction (DE), and first- or second-order Savitzky–Golay derivatives, specifically the first-order derivative with 4 smoothing left- and right-side points and first polynomial order (1,4,4,1), and the second-order derivative with 5 smoothing left- and right-side points and second polynomial order (2,5,5,2). Combinations of these treatments (SNV-DE; SG 1,4,4,1-SNV; SG 1,4,4,1-SNV-DE; SG2,5,5,2-SNV; and SG 2,5,5,2- SNV-DE) were tested for model development. The noise present at both ends of the spectra was removed by trimming the spectral range to 420–2300 nm.

#### 2.4.2. NIR Quantitative Models for Quality Traits

Partial least square regression (PLSR) models were used for quantitative prediction of the analyzed meat quality traits. PLSR models were constructed using the nonlinear iterative partial least squares (NIPALS) algorithm, with the performance models evaluated using leave-one-out full internal cross-validation. The goodness of fit of predictive models was assessed, selecting the model that had the optimal combination of the greatest R^2^_CAL/CV_, the lowest RMSE_CAL/CV_, and the lowest number of PLS latent variables (LVs) considered to be optimal with this result validated using the explained variance test.

Moreover, residual predictive deviation (RPD) and the range error ratio (RER) were calculated. RPD was calculated as the ratio of standard deviation (SD) to the REMCV or RMSEP of a given trait. The RER value was calculated as the ratio between range (concentration amplitude of an analyte) and the RMSE_CV_ or RMSEP of the trait. Finally, external validation was also carried out with the validation sample set and the coefficient of determination of validation (R^2^_VAL_), minimum root mean square error of prediction (RMSEP), and standard error of prediction (SEP) were calculated.

#### 2.4.3. NIR Classification Models for Fresh or Spoiled Pork

In order to test the NIR spectroscopy potential for discrimination between fresh vs. spoiled pork fillets, the commonly established acceptable limits of microbial spoilage [22] were fixed as follows:

- Total viable counts: fresh (acceptable for consumption) if <6.69 log cfu/g; spoiled (unacceptable for consumption) if >6.69 log cfu/g.
- *Enterobacteriaceae* counts: fresh if <4 log cfu/g; spoiled if >4 log cfu/g.
- Lactic acid bacteria counts: fresh if <6.69 log cfu/g; spoiled if >6.69 log cfu/g.

Discriminant analysis by means of partial least square regression discriminant analysis (PLS-DA) was performed using Unscrambler^®^ software (v. 9.8 2008, CAMO, Trondheim, Norway). The independent variable (X) was the spectrum of each sample, while the dependent variable (Y) was a categorical variable defined by the analyst (dummy variables) coding each class numerically. In this study, with two categories, fresh and spoiled, samples belonging to the fresh class were described by the dependent vector [1, 0] and samples belonging to the spoiled class by the vector [0, 1]. A value of 1 was assigned when the sample belonged to the class and 0 when it did not. The optimum number of PLS factors (LVs) for the models was selected by leave-one-out cross-validation.

Discriminant models were evaluated according to the percentage of samples correctly classified during calibration development, cross-validation, and external validation. Sensitivity (SE) indicates the proportion of samples belonging to one specific group that are correctly identified, specificity (SP) refers to the correct identification of samples that do not belong to that group, and accuracy (A) represents the proportion of true results (both true positive and true negative) in the selected population.

The results of SE, SP, and A of calibration, cross-validation, and external validation for TVC, *Enterobacteriaceae* and LAB in the fresh and spoiled categories are expressed as percentages. The Matthews correlation coefficient (MCC) was also calculated, which represents the correlation between predicted and real categories [23,24].

### 2.5. Statistical Analysis

The statistical analysis of meat quality traits data was performed with SPSS v22.0 (SPSS Inc., Chicago, IL, USA). Data of meat quality traits (pH, color, WBSF, and TBARS) were scrutinized for data entry errors and outliers were detected by boxplot. The normality of variables was tested by a Kolmogorov–Smirnoff test. The effects of MAP type (MAP), post-mortem time (T), and their interaction (MAP × T) were analyzed as fixed factors with the animal as covariate using a general linear model. Significant differences among post-mortem times were evaluated by Tukey’s post hoc test (or Games–Howell when the variances were not homogeneous) at a significance level of *p* ≤ 0.05.

## 3. Results and Discussion

### 3.1. Effect of Storage Conditions on Meat Quality Traits and Microbial Loads

Table 1 shows the effects of MAP type, storage time, and their interaction on the analyzed meat quality traits. The MAP type was found to affect TVC and *Enterobacteriaceae* loads, WBSF, TBARS, and color traits (a*, b*, C*, and h*) (*p* < 0.05). On the other hand, storage time after packing only affected microbiological parameters (*p* < 0.001) and meat color traits (a*, b*, C*) (*p* < 0.01). The interaction was significant only for color traits a*, b*, and C*.

**Figure 1:**
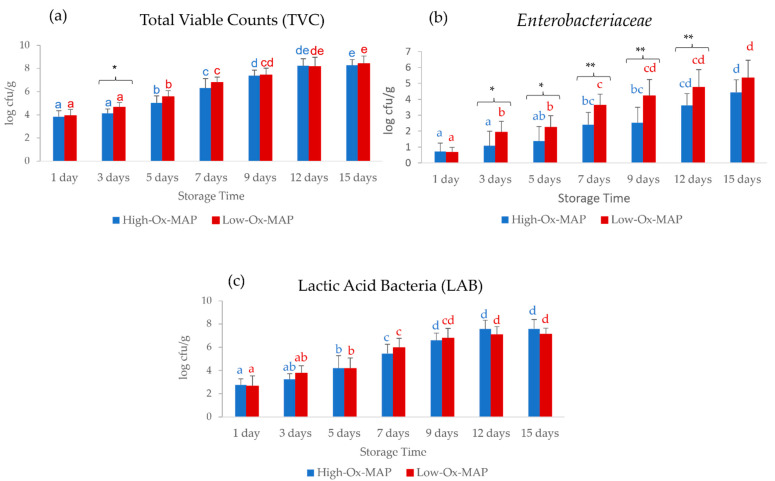
Storage time evolution of (**a**) total viable counts (TVC) (log cfu/g), (**b**) *Enterobacteriaceae* (log cfu/g), and (**c**) lactic acid bacteria (LAB) (log cfu/g) in pork fillets in MAP trays under High-Ox-MAP (30% O_2_/40% CO_2_/30% N_2_, blue) and Low-Ox-MAP (5% O_2_/20% CO_2_/75% N_2_, red) (mean ± standard deviation). Different letters indicate significant differences between storage time at *p* < 0.05. Asterisks indicate significant differences between MAP types at the same storage time. * *p* < 0.05, ** *p* < 0.01.

Figure 1 shows a similar increase (*p* < 0.001) over the storage time for both MAPs, with small differences in microbial loads due to MAP type. In the case of TVC, the differences between MAP types were significant at 3 days of storage, with higher values for meat stored under Low-Ox-MAP (*p* < 0.05). In the case of *Enterobacteriaceae*, higher bacterial loads were found in Low-Ox-MAP at 3, 5, 7, and 9 days (*p* < 0.05) and 12 days (*p* < 0.01) of storage. Bacteria belonging to the *Enterobacteriaceae* family are good indicators of the hygienic condition of raw meat and have facultative anaerobic characteristics. These results show that the use of modified atmosphere packaging exerts effective inhibitory action, due to the addition of active concentrations of carbon dioxide (at least 20%), which results in acidification of the substrate (with formation of carbonic acid), to which enteric bacteria are particularly sensitive. The lower quantity of CO_2_ in Low-Ox-MAP may explain the faster bacterial growth [25,26].

**Figure 2:**
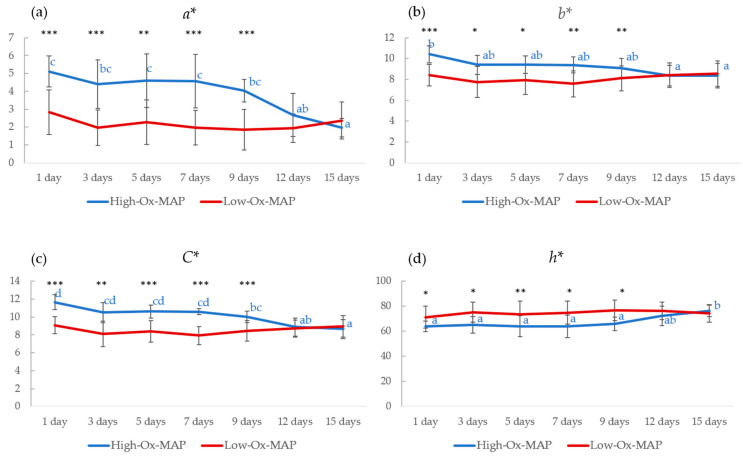
Storage time evolution of (**a**) a*, (**b**) b*, (**c**) C*, and (**d**) h* in pork trays under High-Ox-MAP (30% O_2_/40% CO_2_/30% N_2_, blue) and Low-Ox-MAP (5% O_2_/20% CO_2_/75% N_2_, red) (mean ± standard deviation). Different letters (in blue for High-Ox-MAP) indicate significant differences (*p* < 0.05). Asterisks indicate significant differences between High-Ox-MAP and Low-Ox-MAP at the same storage time. * *p* < 0.05, ** *p* < 0.01, *** *p* < 0.001.

Color attributes, except L*, were significantly affected by MAP, storage time, and their interaction (Figure 2). It is important to note that MAP type produced significant differences for a*, b*, C*, and h*, with higher values for meat packaged under High-Ox-MAP from the beginning of the storage period to 9 days post-mortem for a*, b*, and C*. Subsequently, color attributes were found to be similarly independent of MAP type, probably due to the exhaustion of oxygen and the progression of meat oxidation. No significant difference in color attributes along storage time were seen in Low-Ox-MAP. In High-Ox-MAP, redness (a*), an important factor in the desired pink color in pork, and yellowness (b*), important in determining the onset of brown pigmentation, followed a similar trend with high values at the beginning and decreasing gradually as storage time progressed. Similar results were previously found in beef [27] and pork [28], suggesting that meat stored in high-oxygen packaging had a desirable red color at the beginning of storage, after which some browning occurs.

**Figure 3:**
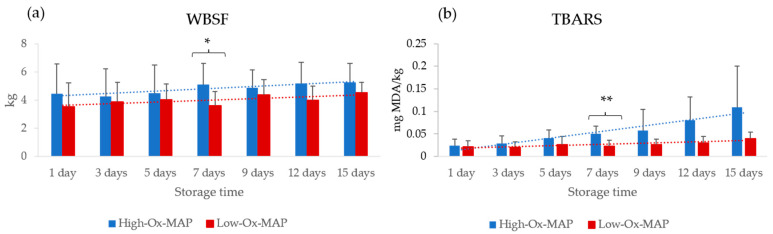
Storage time evolution of (**a**) Warner–Bratzler shear force (WBSF; kg) and (**b**) thiobarbituric acid reactive substances (TBARS; mg malonaldehyde/kg muscle) (mean ± standard deviation) in pork trays under High-Ox-MAP (30% O_2_/40% CO_2_/30% N_2_, blue) and Low-Ox-MAP (5% O_2_/20% CO_2_/75% N_2_, red). * *p* < 0.05, ** *p* < 0.01.

In the case of WBSF and TBARS, MAP types showed significant differences only at 7 days of storage, with higher values for both parameters in pork stored under High-Ox-MAP (Figure 3). A higher O_2_ content in MAP has been previously related to meat quality deterioration through lipid and protein oxidation. In fact, the TBARS index showed an increase (*p* < 0.001) during meat storage and reached a higher level in High-Ox-MAP from day 5 onwards (Figure 3).

High-Ox-MAP trays contain higher percentages of CO_2_, which prevents microbial contamination, and O_2_, which promotes a bright red color that is attractive to consumers. However, high O_2_ levels also promote oxidative changes in meat lipids and proteins, which can negatively affect meat quality, including flavor stability and tenderness [27]. The results found in this study are in agreement with the results of Kim et al. [29], showing that modified atmosphere packaging with high levels of oxygen encourages lipid oxidation, thus increasing TBARS. It has also been previously described that a high level of O_2_ can induce oxidative phenomena and intermolecular cross-linking, which subsequently decreases tenderness and juiciness of meat [30].

### 3.2. NIR Quantitative Models for Quality Traits

Descriptive statistics of meat quality traits analyzed for the calibration and validation datasets are shown in Table 2. Combined data from the different MAP types and storage times were used for calibration development, as the combination may contribute to increasing the variability of quality traits favored in the development of predictive models. In addition, this allows the generation of more generic calibration models that can be used independently of the MAP type used for preserving pork fillets.

**Figure 4:**
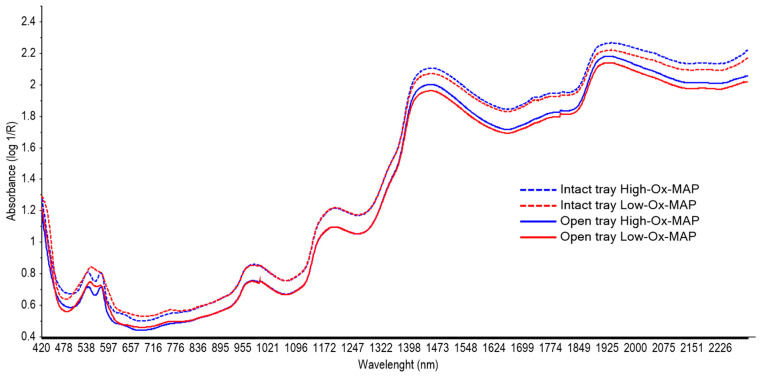
Mean VIS-NIR absorbance (log1/R) spectra (350–2500 nm) of pork meat samples in High-Ox-MAP (blue) or Low-Ox-MAP (red) taken through film on tray (dotted line) of directly on meat (solid line).

Figure 4 shows the mean absorbance spectra collected on the pork fillets taken through the barrier film of intact trays (dotted line) or directly on the meat surface (solid line) in High-Ox-MAP (blue) and Low-Ox-MAP (red). The shape of the spectra collected with and without the barrier film was very similar; however, higher absorbance intensity was found for the spectra taken through the film before opening the trays.

In general, several broad spectral bands could be identified in the VIS-NIR region in averaged spectra of 415, 538, 575, 760, 982, 1195, 1450, and 1925 nm, which are the typical spectral signatures of meat samples characterized by protein, lipid, water, and pigment vibrational signal areas. These features were similar to those previously reported in beef [31,32], chicken [33], and pork [34,35,36]. Bands at the VIS region (415, 538, and 575 nm) may be related to the redox state of myoglobin, responsible for meat color [36,37,38]. In fact, an important difference between High- and Low-Ox-MAP can be observed in the spectral shape of this region, showing two clear bands in the spectra from High-Ox-MAP pork fillets (540 and 580 nm) that were less pronounced in Low-Ox-MAP; this could be due to the promotion of oxidation in High-Ox-MAP. Further, the band shown at 760 nm is related to an absorption band of myoglobin oxidation [37]. The band at 982 nm and the two broad peaks at 1450 and 1925 nm are related to different overtones of the O-H bonds related to water content. The band around 1195 nm is the second overtone of C-H bonds related to meat fat [36].

Results of calibration, cross-validation, and validation of the best models for predicting pork quality traits obtained with spectra taken through the film barrier and directly on the meat surface are shown in Table 3 and Table 4, respectively. It can be observed that the models generated by taking the spectra both through the film on intact trays and on the meat after opening the trays had similar results. In both cases, the best NIR prediction models were obtained for color and microbiology loads. The number of LVs considered by the PLS regressions to build the calibration equations ranged from 4 to 12, which can be considered adequate to avoid overfitting, which would lead to unlikely optimistic predictions. This number is similar to values reported in beef [11], chicken [33], and pork [39,40].

Regarding the best mathematical treatments, SNV was the best for predicting pH, WBSF, a*, b*, C*, h*, TVC, *Enterobacteriaceae*, and LAB in intact trays. In open trays, the best results were obtained by applying the first derivative of Savitzky–Golay with four smoothing points (right and left) for pH, WBSF, TBARS, and h*, and the second derivative with five smoothing points (right and left) for TVC and *Enterobacteriaceae.*

Meat has very heterogeneous characteristics; its structure and composition vary not only between different muscles, but also within the same muscle, and are also affected by time and storage conditions during maturation (temperature, humidity, light, oxygen, etc.) [11,12]. Due to this important variability of meat, results of physical parameters from reference models are also subject to significant variations, which affects the predictive ability of NIR technology [12]. In fact, the quantitative prediction of pH and WBSF in this study was poor. The results obtained for pH showed unreliable predictions both in intact trays (R^2^ = 0.199 and SEP = 0.187) and after opening the trays (R^2^ = 0.483 and SEP = 0.152), similar to previous studies [41,42,43]. However, some studies on intact pork loin [39,40,44] achieved NIR models suitable for rough screening of pH (R^2^_CV_ > 0.62 and SEP < 0.1). To our knowledge, the best results found for VIS-NIR prediction of pH in pork were those reported by Liao et al. [45], with R^2^_CV_ = 0.82 and RMSECV = 0.10 on fresh pork using a prototype of a charge-coupled device (CCD) spectrometer (USB4000-Vis–NIR, Ocean Optics, USA), equipped with a linear CCD detector array (Toshiba TCD1304AP, Japan) in the spectral range of 350–1100 nm.

WBSF prediction also failed to meet the analytical requirements in any case, with coefficient of determination in validation (R^2^_VAL_) of 0.209) on intact trays and 0.252 and SEP over 0.4 after opening the trays. In most of the reviewed literature, the predictive ability of WBSF in intact pork was also poor, making it too weak for industrial applications [35,41,46]. However, Balage et al. [39], whose results for quantitative WBSF prediction were very similar to ours (R^2^_CAL_ = 0.48; R^2^_CV_ = 0.30 and R^2^_VAL_ = 0.25; SEP = 5.51 N and RPD = 1.2), were able to develop classification models that correctly categorized intact pork samples by tenderness (tender or tough, based on WBSF values) with validated 72% accuracy. Therefore, the capability of NIR for screening tender samples should be further explored.

Lipid oxidation is a prominent cause of freshness loss and quality deterioration of muscle foods. TBARS is an important oxidative parameter that can reflect the degree of lipid oxidation [47]. The capacity of NIR to predict TBARS in the present study was poor, with R^2^_CAL_ of 0.615 and 0.528 for spectra taken on intact and open trays, respectively, and RPD_VAL_ under 1.2 in both cases. The poor results obtained for TBARS prediction may be due to the different oxygen exposure of pork fillets within the trays, as the top ones may be more exposed than the rest. In addition, TBARS values vary with location due to the heterogeneity of pork, and this may lead to NIR being unable to predict this parameter accurately. In contrast, Xiong et al. [46] showed the potential of hyperspectral imaging for rapid prediction of TBARS content in chicken meat during refrigerated storage, with regression coefficient in prediction of 0.944 and RMSEP of 0.081.

The results for the prediction of color parameters showed differences depending on how the spectra were taken.

In the case of calibration models generated with the spectra collected through the barrier film of intact trays, the best calibration models were for a* and h*, with R^2^_CAL_ = 0.861/0.922, RER_CV_ = 10.671/10.710, and RPD_CV_ = 2.271/2.217, respectively. In validation, these statistics decreased slightly to R^2^_VAL_ = 0.770/0.789, RER_VAL_ = 7.529/8.289, and RPD_VAL_ = 2.142/2.236, but the values can still be considered good for analytical purposes.

When the models were generated with the spectra collected directly on the pork fillets after opening the trays, the R^2^_CAL_ were 0.878, 0.869, 0.822, and 0.815 for L*, a*, C*, and h* and slightly lower, 0.74, for b*. However, in validation, the best results were obtained for L* (R^2^_VAL_ = 0.869, RER_VAL_ = 11.88, and RPD_VAL_ = 2.85) and b* (R^2^_VAL_ = 0.814, RER_VAL_ = 9.42, and RPD_VAL_ = 2.38). Similar results were reported by Balage et al. [39] for intact pork samples with VIS-NIR spectroscopy performed at 400 to 1395 nm, showing R^2^_CAL_ = 0.88, 0.82, and 0.80; R^2^_VAL_ = 0.77, 0.84, and 0.81; and RPD_VAL_ = 2.3, 2.2, and 2.1 for L*, a*, and b*, respectively. Other studies using VIS-NIR on intact pork samples showed very similar coefficients of determination on validation over 0.77 [40,43,44], but in most of them, RPD_VAL_ was low, ranging from 1.4 to 2, meaning that these equations are promising but should be improved. However, it is important to note that among the studies, this is the first to predict color attributes through the barrier film of the trays.

With respect to the prediction of microbiological parameters, the best calibration models for pork loin quality attributes in the intact trays (with the film) were obtained when SNV and SNVD pre-treatments were applied, while for the opened trays the best results were obtained after SNVD and SG 2,5,5,2 pre-treatment. These pre-treatments have been extensively applied to meat spectra due to their ability to increase spectral resolution, as they can help to separate overlapping absorption bands and remove baseline shifts.

In general, the R^2^_CAL_ values obtained were slightly higher for the models developed after opening the trays (without the film interaction), reaching values over 0.89; however, RMSEC was high, with values around 0.9 log cfu/g, for the three microbiological parameters analyzed, TVC, *Enterobacteriaceae*, and LAB. Looking at the validation results, the goodness of fit of the models decreased to R^2^_VAL_ values of 0.694 for TVC, 0.752 for *Enterobacteriaceae*, and 0.682 for LAB; RPD ranged from 1.858 and 2.161; and SEP was over 0.891 log cfu/g. Horváth et al. [16] obtained better results using diffuse reflectance (1000–2500 nm) for the prediction and evaluation of bacterial contamination in fresh pork, with R^2^_CAL_ = 0.977 and RMSEC = 0.438 log cfu/g. In addition, Atanassova and Stoyanchev [48] achieved similar results on chilled pork loin for prediction of total bacterial count by diffuse reflectance (900–1700 nm), with R^2^cal = 0.86 and SEC = 0.52 log cfu/g. However, none of these studies did real validation, just cross-validation. To our knowledge, the best results obtained in quantitative prediction of microbiology in pork were those from Barbin et al. [22]. These authors developed a system based on NIR hyperspectral imaging (900–1700 nm) to determine the TVC and psychrotrophic plate count (PPC) in refrigerated pork during storage and reported R^2^_VAL_ values of 0.86 and 0.89 for the log TVC and log PPC, respectively.

In the case of the models developed with the intact trays (through the film barrier, before opening the trays), the results were very similar to those obtained after opening the trays. The intact tray models showed R^2^_VAL_ values of 0.776 for TVC, 0.738 for *Enterobacteriaceae*, and 0.725 for LAB, with RPD around 2 and SEP over 0.792 log cfu/g for the three parameters. A similar study by Prado-Marron et al. [2] used a portable NIR system, the Polychromix Phazir™ (Wilmington, MA, USA) to estimate the microbiological parameters in sliced pork on intact MAP trays before opening the package. However, they did not find satisfactory results (R^2^cal ranging between 0.19 and 0.65 and SECV around 1 log cfu/g). The differences between the results obtained in these two studies may be due to the difference in the NIR equipment used. The LABSPEC has a wider range than the Polychromix Phazir (350 to 2500 nm vs. 1600 to 2400 nm) and a larger scanning area (2 cm vs. 0.5 cm wide). According to Hoving-Bolink et al. [49], a large scanning area is needed to reduce the effects of variability in pork and to improve the prediction equations.

Similar results to the ones found in the present study were the ones reported by Grau et al. [14], who showed the potential of a nondestructive visible and short-wavelength near-infrared (SW-NIR) spectroscopy method for monitoring the freshness of sliced and packaged chicken breast. This demonstrates that VIS-NIR spectroscopy has considerable potential as a technique for monitoring food safety and spoilage, with the advantage that it can be used through plastic food packaging. Despite these preliminary results, a larger number of samples will be required to improve the prediction accuracy of our quantitative models.

### 3.3. Classification and Validation of PLS-DA Models for Discriminating Fresh and Spoiled Pork Loin Packed in MAP Trays

Table 5 shows the best fitting discriminant models, developed by means of PLS-DA, for TVC, *Enterobacteriaceae* and LAB in fresh vs. spoiled pork fillets on MAP trays with or without film [22]. Results of calibration, cross-validation, and validation and the mathematical pre-treatment applied in each case are indicated in the table.

In this work, sensitivity indicates the ability to identify fresh pork loin samples that are suitable for consumption, while specificity indicates the ability to identify samples that have high bacterial spoilage and are therefore not suitable for consumption. These models provided very high discriminant ability, showing sensitivity, specificity, and accuracy over 90% in most cases (Table 5). Accuracy refers to the capacity of a model to make a correct classification of samples. However, when the amount of data among categories is unbalanced, it can be misleading. Thus, in this study, we used MCC, which needs good predictions for all categories to reach high values. An MCC value of 1 indicates perfect prediction, and −1 indicates total disagreement between the prediction and true values, and 0 means no better than random prediction [50]. All MCC values were over 0.73, therefore we could assume good predictions for all categories. The best models were those based on TVC and LAB loads without the film (fresh: < 6.69 log cfu/g; spoiled: > 6.69 log cfu/g), showing an MCC values of 0.816 and 1, respectively, and specificity of 100% for the validation samples. This means that all samples that are spoiled and therefore unacceptable for consumption will be correctly identified.

The results obtained through the film barrier were very similar, with MCC values between 0.8 and 1 and specificity over 90%, indicating that NIR can be very useful for on-site control of the final product on store shelves. Previous work by Atanassova and Stoyanchev [51] on chilled pork loin stored at 6 °C for 9 days until spoilage showed similar results to those in this work. They considered meat samples to be fresh or semi-fresh when the total bacterial count values were lower than 6.0 log cfu/g and spoiled when the total bacterial counts were greater than 6.1 log cfu/g. Their results showed correct classification of 96.96% of fresh meat samples and 97.62% of spoiled meat. They found differences between the second-order derivative absorbance spectra of fresh and spoiled meat at 995, 1060, and 1585 nm, related to the absorption of O–H water group or water interacting with protein [52]; 1470 and 1535 nm, related to the vibration of N–H group from secondary amine; 1102, 1222, and 1342 nm, which could be connected to the C─H group; and 1164 to 1176 nm, with a combination of C–H and C–C vibrations. Bacteria, either pathogenic or nonpathogenic, use mainly proteins and carbohydrates from meat structures to maintain vital functions such as growth and biomass augmentation. The informative NIR bands obtained strongly suggest that the differences in NIR spectra between fresh and spoiled meat could be associated with the presence of proteins, free amino acids, amines, or nitrogen-bearing substances and their interactions with water. Hence, rather than exclusively measuring the presence of bacteria per se on the muscle surface, VIS-NIR correlates the extent of biochemical changes in meat to microbial spoilage, allowing us to discriminate between fresh and spoiled samples with qualitative VIS-NIR diffuse reflectance models.

## 4. Conclusions

In the present study, the capability of a portable VIS-NIR tool for on-site freshness detection of pork loin fillets in modified atmosphere packaging (MAP) was analyzed. Quantitative models for predicting quality traits and discriminant models for classifying fresh and spoiled samples were constructed with the intact trays, collecting spectra through the film cover or directly on the pork fillets after opening the trays. Promising predictions for color and microbiology parameters were achieved both with and without the film interaction; however, the best models were those obtained for classifying fresh and spoiled meat. It is pertinent to emphasize that the results obtained in this study for packaged pork represent an important advance for the meat industry. Although the prediction accuracy was not perfect (with a prediction error greater than 0.5 log cfu/g), the use of this VIS-NIR tool on intact trays allows for the non-destructive monitoring of bacterial contamination in a much larger proportion of samples compared to the reference methods. Therefore, implementing this tool will improve product quality control at the production and commercial distribution levels. However, to use this technology for real control in the meat production chain, a considerably higher number of samples should be included in the models to increase the prediction accuracy.

To summarize, these results show that VIS-NIR spectral information and multivariate techniques represent an efficient, rapid, and noninvasive tool that could be used for on-site control of pork freshness and detection of spoilage, enabling the analysis of large numbers of samples throughout commercial distribution and in real-time decision making.

## Figures and Tables

**Table 1:** *P*-values for effect of modified atmosphere type (MAP), storage time (T), and their interaction (MAP × T) on microbiological and meat quality traits.

	MAP	T	MAP × T
TVC (log cfu/g)	0.021 *	0.000 ***	0.547
*Enterobacteriaceae* (log cfu/g)	0.000 ***	0.000 ***	0.154
BAL (log cfu/g)	0.949	0.000 ***	0.264
pH	0.554	0.122	0.529
WBSF (kg)	0.002 **	0.506	0.983
TBARS (mg MDA/kg)	0.021 *	0.072	0.320
L*	0.158	0.419	0.997
a*	0.000 ***	0.000 ***	0.000 ***
b*	0.000 ***	0.069	0.005 **
C*	0.000 ***	0.000 ***	0.000 ***
h*	0.000 ***	0.009 *	0.055

MAP, modified atmosphere packaging; T, storage time; MAP × T, interaction between MAP and time; TVC, total viable counts; LAB, lactic acid bacteria; WBSF, Warner–Bratzler shear force; TBARS, thiobarbituric acid reactive substances; MDA: malonaldehyde; L*, lightness; a*, redness; b*, yellowness; C*, chroma; h*, hue. *p*-values in bold are significant at * *p* < 0.05, ** *p* < 0.01, *** *p* < 0.001.

**Table 2:** Descriptive statistics of meat quality traits of calibration and validation datasets.

	Calibration (*n* = 106)						Validation (*n* = 20)					
	Max	Min	Range	Mean	SD	CV (%)	Max	Min	Range	Mean	SD	CV (%)
pH	6.84	5.29	1.55	5.72	0.23	4.09	6.3	5.42	0.87	5.73	0.21	3.70
WBSF (kg)	8.9	1.44	7.46	4.32	1.56	36.1	6.96	2.64	4.32	4.06	1.06	26.1
TBARS (mg MDA/kg)	0.27	0	0.27	0.04	0.03	71.43	0.07	0.009	0.06	0.03	0.02	54.29
L*	61.75	47.58	14.18	55.36	3.37	6.09	61.37	49.63	11.74	55.48	2.81	5.07
a*	7.36	0.02	7.34	2.89	1.53	53.05	6.75	0.76	5.98	2.99	1.70	56.86
b*	11.58	4.58	7.00	8.58	1.29	15.12	10.96	6.27	4.69	8.65	1.19	13.74
C*	12.70	5.27	7.43	9.20	1.42	15.47	12.63	6.64	5.99	9.3	1.49	15.99
h*	89.9	47.65	42.25	71.9	8.81	12.26	85.89	54.17	31.71	71.74	8.55	11.92
TVC (log cfu/g)	9.25	3.04	6.21	6.37	1.74	27.36	9.12	3.16	5.96	6.19	1.93	31.10
*Enterobacteriaceae* (log cfu/g)	7.23	0	7.23	2.82	1.66	58.93	6.38	0.69	5.68	3.03	1.86	61.39
LAB (log cfu/g)	9.03	1	8.03	5.45	1.91	35.11	7.42	2.24	5.18	4.96	1.73	34.85

Max, maximum; Min, minimum; SD, standard deviation; CV, coefficient of variation; WBSF, Warner–Bratzler shear force; TBARS, thiobarbituric acid reactive substances; MDA, malonaldehyde; L*, lightness; a*, redness; b*, yellowness; C*, chroma; h*, hue; TVC, total viable counts; LAB, lactic acid bacteria.

**Table 3:** Best-fitting NIR prediction equations (cross and external validation statistics) for main quality traits of pork fillets on intact MAP trays and spectral collection through film barrier.

Intact Trays	Treat.	*n*	LVs	RMSE_CAL_	R^2^_CAL_	RMSE_CV_	R^2^_CV_	RER_CV_	RPD_CV_	RMSE_P_	R^2^_VAL_	SEP	RER_VAL_	RPD_VAL_
pH	SNVD	101	11	0.097	0.772	0.170	0.312	7.618	1.203	0.185	0.199	0.187	4.730	1.148
WBSF (kg)	SNVD	98	4	1.14	0.380	1.266	0.251	5.882	1.151	0.912	0.209	0.926	4.728	1.156
TBARS (mg MDA/kg)	SNVD	100	9	0.025	0.615	0.036	0.260	7.491	1.162	0.025	0.02	0.026	2.610	0.775
L*	SG 1,4,4,1	97	4	1.236	0.858	1.980	0.645	7.160	1.671	1.516	0.673	1.547	7.745	1.855
a*	SNVD	101	6	0.577	0.861	0.688	0.807	10.671	2.271	0.795	0.770	0.750	7.529	2.142
b*	SNVD	102	8	0.634	0.722	0.837	0.525	7.859	1.445	0.726	0.606	0.737	6.460	1.636
C*	SNV	96	8	0.619	0.788	0.867	0.594	7.860	1.563	0.841	0.662	0.843	7.121	1.767
h*	SNVD	102	9	2.421	0.922	3.945	0.798	10.710	2.217	3.826	0.789	3.796	8.289	2.236
TVC (log cfu/g)	SNV	98	7	0.753	0.796	0.942	0.688	6.517	1.783	0.888	0.776	0.891	6.715	2.170
ENT (log cfu/g)	SNVD	98	6	0.725	0.787	0.851	0.712	7.117	1.856	0.927	0.738	0.932	6.132	2.009
LAB (log cfu/g)	SNV	106	8	0.827	0.810	1.044	0.704	7.694	1.830	0.882	0.725	0.792	5.874	1.960

Treat., mathematical pre-treatment; n, number of samples; LVs, latent variables; RMSE_CAL_/RMSE_CV_/RMSE_P_, root mean square error of calibration/cross-validation/validation; R^2^_CAL_/R^2^_CV_/R^2^_VAL_, coefficient of determination of calibration/cross-validation/validation; SEP, standard error of prediction; RER_CV_/RER_VAL_, error range of cross-validation/validation ratio; RPD_CV_/RPD_VAL_, performance deviation of cross-validation/validation ratio, ABS: absorbance spectra (log 1/R); SNV, standard normal variate; SNVD, standard normal variate and detrend; SG 1,4,4,1, Savitzky–Golay first-order derivative, 4 smoothing left-side points, 4 smoothing right side points, 1 polynomial order; WBSF, Warner–Bratzler shear force; TBARS, thiobarbituric acid reactive substances; MDA, malonaldehyde; L*, lightness; a*, redness; b*, yellowness; C*, chroma; h*, hue; TVC, total viable counts; ENT, *Enterobacteriaceae*; LAB, lactic acid bacteria.

**Table 4:** Best-fitting NIR prediction equations (cross and external validation statistics) for main quality traits of pork fillets after opening MAP trays and spectral collection directly on meat surface.

Open Trays	Treat.	n	LVs	RMSE_CAL_	R^2^_CAL_	RMSE_CV_	R^2^_CV_	RER_CV_	RPD_CV_	RMSEP	R^2^_VAL_	SEP	RER_VAL_	RPD_VAL_
pH	SG 1,4,4,1	106	10	0.095	0.833	0.164	0.508	9.421	1.426	0.148	0.483	0.152	5.912	1.435
WBSF (kg)	SG 1,4,4,1	98	4	0.976	0.532	1.076	0.444	6.917	1.336	0.888	0.252	0.882	4.856	1.187
TBARS (mg MDA/kg)	SG 1,4,4,1	86	9	0.013	0.528	0.016	0.329	6.907	1.261	0.017	0.154	0.017	3.848	1.141
L*	ABS	97	5	1.126	0.878	1.247	0.854	11.368	2.604	0.988	0.869	1.013	11.884	2.846
a*	ABS	99	5	0.540	0.869	0.589	0.848	11.330	2.559	0.894	0.709	0.806	6.695	1.905
b*	SNVD	106	5	0.657	0.740	0.718	0.695	9.753	1.806	0.498	0.814	0.506	9.418	2.386
c*	SNV	106	9	0.597	0.822	0.814	0.676	9.127	1.748	0.750	0.731	0.714	7.985	1.981
h*	SG 1,4,4,1	106	4	3.763	0.815	4.145	0.780	10.194	2.126	4.682	0.684	4.717	6.774	1.827
TVC (log UF C/g)	SG 2,5,5,2	106	12	0.556	0.897	0.992	0.679	6.260	1.692	1.037	0.694	0.898	5.750	1.858
ENT (log CFU/g)	SG 2,5,5,2	98	11	0.473	0.911	0.940	0.657	6.847	1.700	0.903	0.752	0.925	6.295	2.062
LAB (log CFU/g)	SNVD	95	10	0.587	0.900	0.963	0.735	8.341	1.936	0.800	0.682	0.541	6.476	2.161

Treat., mathematical pre-treatment; n, number of samples; LVs, latent variables; RMSE_CAL_/RMSE_CV_/RMSE_P_, root mean square error of calibration/cross-validation/validation; R^2^_CAL_/R^2^_CV_/R^2^_VAL_, coefficient of determination of calibration/cross-validation/validation; SEP, standard error of prediction; RER_CV_/RER_VAL_, error range of cross-validation/validation ratio; RPD_CV_/RPD_VAL_, performance deviation of cross-validation/validation ratio; ABS, absorbance spectra (log 1/R); SNV, standard normal variate; SNVD, standard normal variate and detrend; SG 1,4,4,1, Savitzky–Golay first-order derivative, 4 smoothing left-side points, 4 smoothing right-side points, 1 polynomial order; SG 2,5,5,2, Savitzky–Golay first-order derivative, 5 smoothing left-side points, 5 smoothing right-side points, 2 polynomial order; WBSF, Warner–Bratzler shear force; TBARS, thiobarbituric acid reactive substances; MDA, malonaldehyde; L*, lightness; a*, redness; b*, yellowness; C*, chroma; h*, hue; TVC, total viable counts; ENT, *Enterobacteriaceae*; LAB, lactic acid bacteria.

**Table 5:** Statistics of PLS-DA models for classification of fresh vs. spoiled pork on intact and opened trays.

Total Viable Counts (Fresh/Spoiled)	Intact Trays			Open Trays		
Pre-treatment	SG 2,5,5,2 SNVD			SNV		
	**CAL**	**CV**	**VAL**	**CAL**	**CV**	**VAL**
Sensitivity (%)	97.72	93.18	90	97.72	90.9	80
Specificity (%)	98.2	92.85	90	100	100	100
Accuracy (%)	98	93	90	99	96.11	90
Matthews correlation	0.959	0.858	0.8	0.98	0.922	0.816
*Enterobacteriaceae* (fresh/spoiled)	**Intact trays**			**Open trays**		
Pre-treatment	SNV			SG 2,5,5,2 SNV		
	**CAL**	**CV**	**VAL**	**CAL**	**CV**	**VAL**
Sensitivity (%)	91.89	90.5	100	98.66	97.33	92.85
Specificity (%)	89.47	89.47	100	95	70	83.33
Accuracy (%)	91.39	90.32	100	97.89	91.57	90
Matthews correlation	0.76	0.737	1	0.936	0.733	0.761
Lactic Acid Bacteria (fresh/spoiled)	**Intact trays**			**Open trays**		
Pre-treatment	SG 1,4,4,1 SNVD			SG 1,4,4,1 SNV		
	**CAL**	**CV**	**VAL**	**CAL**	**CV**	**VAL**
Sensitivity (%)	98	98	91.66	98	94.23	100
Specificity (%)	86.95	84.78	100	100	97.77	100
Accuracy (%)	92.85	91.83	95	98.96	95.87	100
Matthews correlation	0.86	0.835	0.902	0.979	0.918	1

CAL, calibration; CV, cross-validation; VAL, validation; SNV, standard normal variate; SNVD, standard normal variate and detrend; SG 1,4,4,1, Savitzky–Golay first-order derivative, 4 smoothing left-side points, 4 smoothing right-side points, 1 polynomial order; SG 2,5,5,2, Savitzky–Golay first-order derivative, 5 smoothing left-side points, 5 smoothing right-side points, 2 polynomial orders.

## Data Availability

Available on request.
